# Determination of fruit maturity and its prediction model based on the pericarp index of absorbance difference (*I*_AD_) for peaches

**DOI:** 10.1371/journal.pone.0177511

**Published:** 2017-05-15

**Authors:** Binbin Zhang, Bin Peng, Chunhua Zhang, Zhizhong Song, Ruijuan Ma

**Affiliations:** Institute of Pomology, Jiangsu Academy of Agricultural Sciences/Jiangsu Key Laboratory for Horticultural Crop Genetic Improvement, Nanjing, Jiangsu, China; Wuhan Botanical Garden, CHINA

## Abstract

Harvest maturity is closely related to peach fruit quality and has a very important effect on the fresh fruit market. Unfortunately, at present, it is difficult to determine the maturity level of peach fruits by artificial methods. The objectives of this study were to develop quadratic polynomial regression models using near-infrared spectroscopy that could determine the peel color difference, fruit firmness, soluble solids content (SSC), soluble sugar, organic acid components, and their relationships with the absorbance of chlorophyll (index of absorbance difference, *I*_AD_) in late maturing ‘Xiahui 8’ peach and ‘Xiaguang’ nectarine fruits. The analysis was based on data for fruits at veraison, fruits at harvesting maturity, and all fruits. The results showed that firmness has the highest correlation coefficient with *I*_AD_. Prediction models for fruit maturity were established between firmness and the *I*_AD_ of the two cultivars using the quadratic polynomial regression method. Further variance analysis on the one degree term and quadratic term of each equation showed that every partial regression coefficient reached a significant or extremely significant level. No significant difference was observed between estimated and observed values after regression prediction. The regression equations seem to fit well. Other peach and nectarine varieties were used to test the feasibility of maturity prediction by this method, and it was found that maturity was successfully predicted in all the samples. The result indicated that the *I*_AD_ can be used as an index to predict peach fruit maturity.

## Introduction

In the peach market, the maturity at fruit harvest is always the main factor that restricts its commodity value. Accurately determining peach maturity plays an important role in its timely harvesting, classification, packing, transportation, and the guarantee of commodity quality [1]. As a respiration climacteric fruit, peaches release an increased amount of ethylene during ripening, and gene transcription also varies, which is often regulated by plant hormones [2–4]. During this process, fruit firmness, the composition and ratio of fruit inclusion, and the peel color change accordingly, and the quality related indicators are significantly different in fruits at different maturity levels [5]. Many studies have investigated the peach maturity level using destructive or non-destructive methods [1,6,7]. The correlation between soluble solids content (SSC) and maturity, and regression analysis of fruit SSC and quality indicators in ‘Hujingmilu’ peach, Zhang et al. [1] established prediction equations for firmness and SSC using the quadratic polynomial regression method. Nascimento et al. [8] used near-infrared spectroscopy to investigate peach maturity predictions by the partial least square (PLS) model of the SSC and fruit firmness in low-chilling peach. They created prediction models for SSC and fruit firmness, and established the optimization potential of the model. Matteoli et al. [9] proposed a spectral-based non-destructive method for the classification of peach maturity levels that estimates the firmness of the flesh to classify the maturity level by the reflectance spectra. They used multiple retrieval techniques and the fuzzy classification system, and this method lays the foundation for the automatic classification of peach fruit maturity.

The index of absorbance difference (*I*_AD_) is an indicator that is based on the close relationship between the degradation of chlorophyll and the maturity of the fruit, which is determined by the difference between the absorption at 670 nm and 720 nm using near-infrared spectroscopy. It directly reflects the actual content of chlorophyll a [4]. The non-destructive measurement of *I*_AD_ is not harmful to fruit, the reading is fast and convenient, and it is more desired than the destructive assays, such as firmness and SSC. Therefore, it is highly suitable for fruit quality estimation at the end of the supply chain. Currently, *I*_AD_ predication is carried out mostly on stone fruit trees, such as peach [10,11] and plum [12], etc. Gonçalves et al. [13] performed a non-destructive evaluation on seven peach and five nectarine varieties and found that there was an extremely significant linear regression relationship between the *I*_AD_ value and fruit firmness, and that there were variations among different varieties. They also showed that there was no significant relationship between the *I*_AD_ value and fruit SSC. Lurie et al. [14] collected the *I*_AD_ at harvest of both early and late maturity peach varieties, carried out a non-linear regression analysis of the change in firmness during shelf time, and established the Logistic model of firmness change. They used time resolution reflectance spectroscopy to evaluate the degree of maturity and believed that the measurement of *I*_AD_ at harvest might classify the fruits into various categories based their potential shelf time, which may ensure better fruit quality.

Previous studies mainly used SSC and titratable acid content as internal quality indicators for the prediction of peach maturity by the *I*_AD_ value, but there have been few studies on the effect of composition content and its ratio on maturity. In addition, the relationship between peach maturity and firmness has rarely been investigated by previous studies. In peach production, it is common to choose peach fruits at veraison for long-distance transportation, but fruits at harvesting maturity are more suitable for the fresh fruit market. In this study, we used the peach variety ‘Xiahui 8’ and nectarine variety ‘Xiaguang’ at different maturity points to comprehensively analyze the peach color differences, firmness, internal quality indicators, and pericarp *I*_AD_ value, and tried to establish a prediction model of fruit maturity in order to provide a scientific basis for fruit harvesting time.

## Materials and methods

### Fruit material

The experiment was performed at the peach orchard of Jiangsu Academy of Agricultural Sciences in 2015 using the fruits of the 7a late maturity peach variety ‘Xiahui 8’ and late maturity nectarine variety ‘Xiaguang’ as experimental materials. At fruit maturation, 30 of the developmentally uniform fruits at veraison (maturity degree I) and 30 of fruits that had reached harvesting maturity (maturity degree II) were collected from the central periphery of the tree at 8:00 am on a sunny morning. All the fruits were immediately brought back to the laboratory. Every fruit was numbered. The fruits were split and the middle of the two sides was labeled for each fruit. The *I*_AD_ value, color difference, firmness, and SSC were sequentially determined, and then the flesh of the two sides was cut and homogenized to evaluate the soluble carbohydrate and organic acids using high performance liquid chromatography. The average of the two sides was used as the corresponding indicator for each fruit. The above-mentioned indicators for each fruit were one-to-one matched by ensuring the order of the assays. The tested trees showed moderate growth with an open vase form. They were planted north to south with ridge cultivation, and were managed by regular cultivation practices.

In 2016, 60 fruits for each variety (‘Xiahui 8’, ‘Xiaguang’) were chosen randomly during the ripening process to determine their firmness and *I*_AD_ values. In addition, fruit firmness and the *I*_AD_ values of three peach varieties (‘Xiahui 5’, ‘Baihuashuimi’, and ‘Wanhujing’) and three nectarine varieties (‘Zijinhong 1’, ‘Zijinhong 2’, and ‘Huyou 018’) were determined. For each variety, 30 fruits at veraison and 30 fruits at harvesting maturity were used.

### Index determination

#### Index of absorbance difference

The index of absorbance difference (*I*_AD_) can reflect the status of fruit maturity by measuring the changes in pericarp chlorophyll content, which is calculated as the difference between the absorption at 670 nm and 720 nm within the range of 0–2.2. The value 2.2 represents green, and 0 represents complete maturity [4,15]. The *I*_AD_ value of the pericarp was determined by a DA-Meter (TR Turoni srl, Forlì, Italy).

#### Color

A Color Quest XE (Hunter Lab, Reston, VA, USA) color difference meter was used to evaluate the pericarp brightness (L^*^), where a^*^ represents “-green” to “+red” and b^*^ represents “-blue” to “+yellow”. Then, the chroma (C^*^), hue angle (*h*) [16,17], and a^*^/b^*^ were calculated.

#### Firmness

The firmness with and without the pericarp was determined by a TA-XT Plus texture analyzer (Stable Micro-Systems Texture Technologies Corp, Scarsdale, New York, NY, USA) with a probe diameter of 8 mm, a test depth of 5 mm, and a penetration rate of 1 mm s^−1^.

#### Soluble solids content

Flesh SSC values were measured using a digital, hand-held pocket refractometer PAL-1 (ATAGO, Itabashi-ku, Tokyo, Japan). The SSC was expressed in Brix at 20°C [18,19].

#### Soluble sugar and organic acid components

The sucrose, glucose, fructose, sorbitol, malic acid, quinic acid, and citric acid contents were measured by a Agilent 1100 high performance liquid chromatography (Agilent Technology, Santa Clara, CA, USA) [20]. The total sugar content was the content sum of four kinds of soluble sugars and the total acid content was the total content of four kinds of organic acids. The sugar/acid ratio was calculated from the total sugar and total acid contents.

### Data analysis

In 2015, the average, standard deviation (SD), amplitude, range, and coefficient of variation (CV) for fruits with maturity degrees I and II were calculated for both varieties. The correlations between *I*_AD_ value and the color difference, firmness, SSC, soluble sugar content, and organic acid indicators of the fruits at maturity degrees I and II and for all fruits were analyzed, and a regression analysis was performed on the indicators closely related to the *I*_AD_ value to try and establish the equation for the prediction of peach fruit maturity. Data processing and analysis were carried out using Microsoft Excel 2010 (Microsoft Corp, Redmond, WA) and SPSS (Version 17.0, SPSS Inc., Chicago, IL, USA).

In 2016, both firmness and the *I*_AD_ for every fruit of ‘Xiahui 8’ and ‘Xiaguang’ were used to test the feasibility of the regression equations which were established in this study. Data for firmness and the *I*_AD_ values of other peach and nectarine varieties, respectively, were used to establish the regression equations.

## Results

### Analysis of the variation on the fruit quality indicators

As shown in Tables 1 and 2, the fruit *I*_AD_, color difference, firmness, SSC, sucrose, fructose, sorbitol, citric acid, and total sugar contents of ‘Xiahui 8’ and ‘Xiaguang’ at maturity degree I showed similar trends when compared to those at maturity degree II. The fruit *I*_AD,_ L^*^, b^*^, *h*, firmness with pericarp, firmness without pericarp, and sorbitol and citric acid contents were all lower for maturity degree II fruit than for those at maturity degree I, but the a^*^, C^*^, a^*^/b^*^, SSC, sucrose and total sugar content measurements produced opposite results. Differences were observed in the glucose, malic acid, quinic acid contents, and the total acid and sugar acid ratio between the two varieties at the different maturity degree points. The glucose content and sugar acid ratio of ‘Xiahui 8’ at degree I were lower than at degree II, whereas the malic acid, quinic acid, and total acid contents were higher, but these indicators showed an opposite trend in ‘Xiaguang’. These data indicate that a higher maturity degree results in a lower *I*_AD_ value, lower fruit firmness, a deeper red color, and a higher inclusion content, but the soluble sugar and organic acid compositions varied in the different varieties.

**Table 1:** Variation analysis of the fruit quality indexes for‘Xiahui 8’ peach.

Index	Maturity Degree I					Maturity Degree II				
	Mean	SD	Amplitude	Range	CV	Mean	SD	Amplitude	Range	CV
*I*_AD_	0.53	0.26	0.19–1.17	0.98	48.92	0.12	0.11	0–0.36	0.36	86.69
L^*^	69.36	4.78	61.13–77.68	16.55	6.89	53.25	4.79	42.77–62.08	19.31	9.00
a^*^	15.36	5.71	4.09–24.54	20.46	37.18	31.61	3.14	23.11–36.18	13.07	9.93
b^*^	23.31	1.81	20.14–26.92	6.78	7.76	18.31	1.60	14.71–21.43	6.73	8.73
C^*^	28.93	2.33	24.97–34.52	9.55	8.04	36.65	2.73	31.27–40.89	9.61	7.46
*h*	57.97	11.26	40.80–80.82	40.02	19.43	30.21	3.52	25.44–40.80	15.36	11.66
a^*^/b^*^	0.69	0.29	0.16–1.16	1.00	41.78	1.75	0.21	1.28–2.10	0.82	11.80
Firmness with Pericarp (N)	173.16	15.96	140.24–203.85	63.61	9.22	89.00	36.19	26.60–145.14	118.50	40.66
Firmness without Pericarp (N)	95.59	10.18	72.26–110.95	38.68	10.65	40.71	20.02	5.97–67.32	61.34	49.18
SSC (°Brix)	11.94	1.14	8.75–14.15	5.40	9.57	12.44	1.74	10.00–17.25	7.25	13.95
Sucrose (g kg^–1^)	49.82	8.02	25.89–60.56	34.67	16.11	54.84	9.41	38.74–75	36.26	17.16
Glucose (g kg^–1^)	13.18	1.61	6.39–16.24	9.85	12.22	14.97	1.29	12.39–17.44	5.05	8.61
Fructose (g kg^–1^)	11.87	1.33	9.16–15.54	6.38	11.18	13.42	1.64	10.94–18.33	7.40	12.20
Sorbitol (g kg^–1^)	6.25	1.97	1.18–9.87	8.69	31.49	2.58	1.79	0.58–7.56	6.98	69.33
Malic Acid (g kg^–1^)	2.71	0.45	2.16–4.64	2.48	16.63	2.11	0.22	1.71–2.62	0.91	10.63
Quinic Acid (g kg^–1^)	1.18	0.36	0.75–2.44	1.69	30.83	1.08	0.28	0.56–1.85	1.28	26.18
Citric Acid (g kg^–1^)	0.35	0.13	0–0.55	0.55	35.52	0.02	0.05	0–0.15	0.15	228.50
Total Sugar (g kg^–1^)	81.13	9.81	50.14–95.50	45.36	12.10	85.81	11.48	69.27–111.33	42.06	13.38
Total Acid (g kg^–1^)	4.24	0.78	3.34–7.43	4.09	18.42	3.22	0.41	2.46–4.28	1.82	12.86
Sugar Acid Ratio	19.75	3.86	9.86–27.27	17.41	19.56	27.00	4.08	18.98–34.97	15.99	15.10

SD, standard deviation. CV, coefficient of variation.

**Table 2:** Variation analysis of the fruit quality indexes for ‘Xiaguang’ nectarine.

Index	Maturity Degree I					Maturity Degree II				
	Mean	SD	Amplitude	Range	CV	Mean	SD	Amplitude	Range	CV
*I*_AD_	0.92	0.32	0.30–1.52	1.22	35.17	0.39	0.26	0–1.03	1.03	67.26
L^*^	60.85	4.68	52.02–68.76	16.74	7.69	52.53	4.08	45.48–62	16.52	7.77
a^*^	19.95	7.62	3–34.17	31.17	38.20	31.81	4.50	22.58–39.33	16.75	14.14
b^*^	35.58	4.86	25.67–44.08	18.42	13.67	27.65	3.76	21.21–37.25	16.04	13.59
C^*^	42.18	2.39	36.51–47.52	11.02	5.66	42.75	2.07	37.87–46.63	8.76	4.85
*h*	60.38	11.83	39.84–85.95	46.11	19.59	41.01	7.38	29.73–57.06	27.33	17.99
a^*^/b^*^	0.63	0.31	0.07–1.20	1.13	48.89	1.22	0.30	0.66–1.76	1.10	24.41
Firmness with Pericarp (N)	152.27	28.31	79.91–194.54	114.63	18.59	89.77	40.68	32.80–183.90	151.10	45.32
Firmness without Pericarp (N)	77.48	17.78	38.13–110.06	71.92	22.94	34.42	25.38	5.43–98.48	93.05	73.73
SSC (°Brix)	14.19	2.15	11.30–20.25	8.95	15.13	15.19	2.19	10.10–19.55	9.45	14.40
Sucrose (g kg^–1^)	59.95	8.41	43.51–76.93	33.42	14.02	65.52	11.55	29.46–86.85	57.39	17.64
Glucose (g kg^–1^)	15.46	1.19	12.68–18.05	5.37	7.67	15.20	1.64	10.55–17.49	6.94	10.80
Fructose (g kg^–1^)	13.05	0.94	11.36–15.36	4.01	7.21	14.31	1.45	10.43–16.67	6.24	10.13
Sorbitol (g kg^–1^)	9.82	4.03	4.37–19.24	14.87	41.09	6.17	3.90	0.87–13.40	12.53	63.29
Malic Acid (g kg^–1^)	3.10	0.34	2.47–3.74	1.26	11.04	3.10	0.50	2.22–4.23	2.01	16.04
Quinic Acid (g kg^–1^)	1.64	0.33	1.06–2.43	1.37	20.03	1.80	0.29	1.24–2.20	0.96	16.04
Citric Acid (g kg^–1^)	0.37	0.27	0–1.34	1.34	71.64	0.50	0.27	0–1.04	1.04	54.86
Total Sugar (g kg^–1^)	98.28	12.60	79.86–124.24	44.38	12.82	101.20	14.90	58.31–121.80	63.49	14.72
Total Acid (g kg^–1^)	5.11	0.72	3.90–6.69	2.79	14.16	5.41	0.74	3.80–6.85	3.06	13.64
Sugar Acid ratio	19.46	1.95	15.1–22.95	7.85	10.01	19.01	2.98	10.39–23.63	13.24	15.69

SD, standard deviation. CV, coefficient of variation.

In addition, Tables 1 and 2 show that although manual classification of maturity was performed during fruit harvesting, fruits with the same maturity degree still showed significant differences for many indicators and a relatively large amplitude and CV.

The CVs for fruit *I*_AD_, and sorbitol, quinic acid, and citric acid contents were relatively large in ‘Xiahui 8’ at both maturity degree points. Furthermore, the CVs for a^*^, *h*, a^*^/b^*^, total acid, and the sugar acid ratio in fruits at degree I maturity, and firmness with pericarp and without pericarp in degree II fruit were also large (Table 1). Fruit *I*_AD_, *h*, a^*^/b^*^, firmness with pericarp, firmness without pericarp, and the sorbitol, quinic acid, and citric acid contents of ‘Xiaguang’ at both maturity degree points had relatively high CVs, as did the a^*^ in degree I fruit and sucrose content of degree II fruit (Table 2). Our results suggest that *I*_AD_ is the most sensitive indicator for determining peach maturity. Indicators with relatively high CVs showed a closer relationship with *I*_AD_, and they had more significant effects on fruit maturity.

### Correlation analysis between the quality indicators of various fruit populations and *I*_AD_ value

The correlation analysis between *I*_AD_ value and the indicators for degree I fruits, degree II fruits, and all the collected fruits are shown in Table 3. In ‘Xiahui 8’, significant or extremely significant correlations were observed between *I*_AD_ value and every tested indicator for all the fruits except for quinic acid content. The *I*_AD_ value was significantly or extremely significantly correlated with color difference, firmness, and glucose and sorbitol contents in ‘Xiaguang’, but not with other indicators. The correlation analysis between *I*_AD_ value and the indicators for the three fruit populations for each variety suggested that most indicators, such as L^*^, *h*, a^*^/b^*^, sucrose, quinic acid, total acid, sugar acid ratio, etc., were significantly or extremely significantly correlated with the *I*_AD_ value only at certain maturity degree points and that there was a poor consistency between varieties. The results demonstrated that the correlations between *I*_AD_ value and C^*^, firmness with pericarp, firmness without pericarp, and sorbitol content were significant and extremely significant in the different fruit populations (except for the firmness with or without pericarp and the sorbitol content of ‘Xiahui 8’ degree I and degree II fruit, and the C^*^ of degree I ‘Xiaguang’ fruit), which indicated that these four indicators are more suitable for predicting peach maturity. The absolute value of the correlation coefficient between the firmness with or without pericarp and the *I*_AD_ value was higher than that for C^*^ and sorbitol content, which suggested that fruit firmness is closely related to the maturity determination. The correlation coefficient between firmness with pericarp and without pericarp was 0.966^**^ and 0.955^**^ for ‘Xiahui 8’ and ‘Xiaguang’, respectively. Therefore, it should be possible to establish a maturity prediction model using a regression analysis of firmness data with or without pericarp and the *I*_AD_ value.

**Table 3:** Correlation analysis between the fruit quality indexes and the *I*_AD_ values.

Index	‘Xiahui 8’			‘Xiaguang’		
	Maturity Degree I	Maturity Degree II	All Fruits	Maturity Degree I	Maturity Degree II	All Fruits
L^*^	-0.58^**^	-0.26	0.47^**^	0.08	0.05	0.50^**^
a^*^	0.32	0.44^*^	-0.52^**^	-0.38^*^	-0.30	-0.65^**^
b^*^	0.09	0.03	0.63^**^	0.17	-0.06	0.50^**^
C^*^	0.43^*^	0.43^*^	-0.46^**^	-0.15	-0.63^**^	-0.34^**^
*H*	-0.26	-0.32	0.53^**^	0.36^*^	0.12	0.62^**^
a^*^/b^*^	0.24	0.29	-0.58^**^	-0.31	-0.18	-0.61^**^
Firmness with Pericarp	-0.13	0.79^**^	0.69^**^	0.84^**^	0.90^**^	0.91^**^
Firmness without Pericarp	-0.08	0.61^**^	0.69^**^	0.73^**^	0.83^**^	0.87^**^
SSC	-0.73^**^	-0.24	-0.43^**^	0.36^*^	0.41^*^	0.12
Sucrose	-0.73^**^	-0.17	-0.52^**^	0.24	0.09	-0.07
Glucose	0.03	-0.07	-0.38^**^	0.32	0.31	0.29^*^
Fructose	0.21	-0.12	-0.28^*^	0.24	0.31	-0.14
Sorbitol	-0.57^**^	0.25	0.35^**^	0.47^**^	0.86^**^	0.71^**^
Malic Acid	0.16	0.23	0.56^**^	0.31	0.30	0.21
Quinic Acid	-0.09	-0.36^*^	0	0.54^**^	0.31	0.14
Citric Acid	0.25	0.41^*^	0.72^**^	0.47^**^	0.06	0.04
Total Sugar	-0.68^**^	-0.12	-0.46^**^	0.35	0.36^*^	0.19
Total Acid	0.09	-0.07	0.49^**^	0.57^**^	0.35	0.20
Sugar Acid Ratio	-0.48^**^	-0.06	-0.65^**^	-0.34	0.03	-0.03

Coefficients followed by one (*) and two asterisks (**) are significant at *P* < 0.05 and *P* < 0.01, respectively.

### Regression analysis based on the firmness and *I*_AD_ value

**Fig 1:**
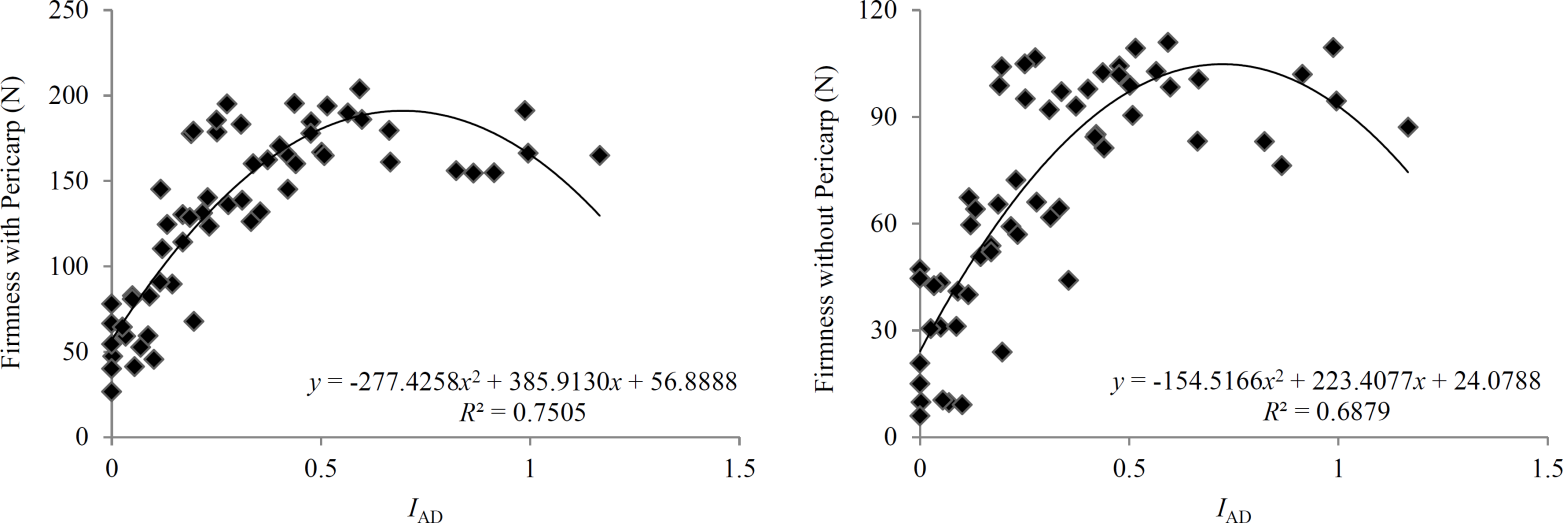
Regression curve between fruit firmness and the *I*_AD_ of ‘Xiahui 8’ peaches.

**Fig 2:**
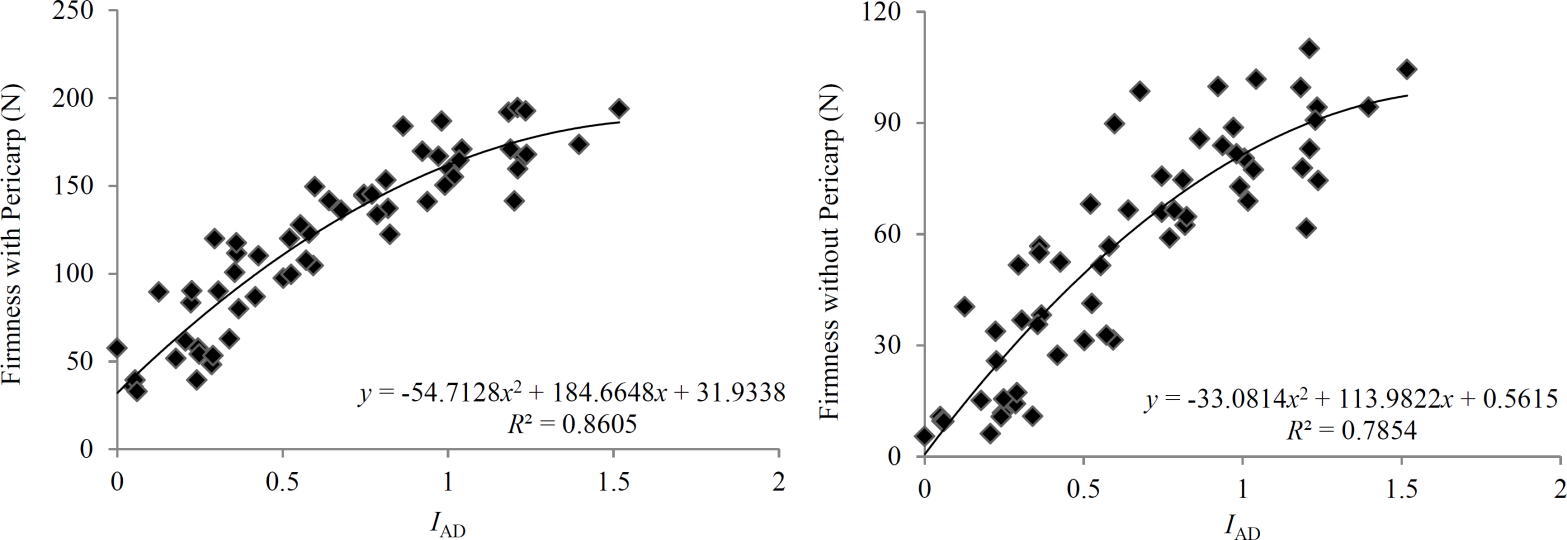
Regression curve between fruit firmness and the *I*_AD_ of ‘Xiaguang’ nectarines.

Linear and quadratic polynomial regressions were performed between the firmness with or without pericarp and the *I*_AD_ value for all fruits of both the ‘Xiahui 8’ and ‘Xiaguang’ varieties, and the results are shown in Table 4, Fig 1 and Fig 2. The *P* value of all the regression models was 0.0001, which indicated an extremely significant regression. The *R*^2^ of the two polynomial regressions was higher than the linear regression for the same indicator and same variety, and its Durbin-Watson statistic value was closer to 2, which suggested that the model was more stable. The variance analysis of each quadratic term and linear term in the quadratic polynomial regression equation showed that the partial regression coefficient of the quadratic term and linear term in the firmness with/without pericarp model for ‘Xiahui 8’ and the firmness with the pericarp model for ‘Xiaguang’ reached a significant level (*P* < 0.01) (Table 5). However, the partial regression coefficients were extremely significant (*P* < 0.01) and significant (*P* < 0.05) for the linear term and quadratic term in the firmness without pericarp model for ‘Xiaguang’, respectively. The regression prediction was not significantly different for the difference between the estimated value and the observed value, which suggested that the regression equation had a good fit.

**Table 4:** Maturity prediction models between fruit firmness and *I*_AD_ value.

Index	Variety	Regression	Maturity prediction model	*R*^2^	*F*	*P*	Durbin-Watson Statistic
Firmness with Pericarp	‘Xiahui 8’	Liner Regression	*y* = 123.3960*x* + 90.9903	0.4758	52.6405	0.0001	1.9073
		Quadratic Polynomial Regression	*y* = -277.4258*x*^2^ + 385.9130*x* + 56.8888	0.7505	85.7284	0.0001	1.9907
	‘Xiaguang’	Liner Regression	*y* = 108.1900*x* + 50.1461	0.8310	285.1840	0.0001	1.7836
		Quadratic Polynomial Regression	*y* = -54.7128*x*^2^ + 184.6648*x* + 31.9338	0.8605	175.7354	0.0001	2.0177
Firmness without Pericarp	‘Xiahui 8’	Liner Regression	*y* = 77.1944*x* + 43.0722	0.4720	51.8410	0.0001	1.7848
		Quadratic Polynomial Regression	*y* = -154.5166*x*^2^ + 223.4077*x* + 24.0788	0.6879	62.8394	0.0001	1.8885
	‘Xiaguang’	Liner regression	*y* = 67.7427*x* + 11.5734	0.7602	183.8180	0.0001	1.8438
		Quadratic Polynomial Regression	*y* = -33.0814*x*^2^ + 113.9822*x* + 0.5615	0.7854	104.2310	0.0001	1.9764

**Table 5:** Variance analysis of the regression coefficients for quadratic polynomial regression.

Index	Variety	Variable	Partial Correlation	*t* test	*P*
Firmness with Pericarp	‘Xiahui 8’	*r*(*y*, *x*)	0.8237	10.9675	0.0001
		*r*(*y*, *x*^2^)	-0.7239	7.9222	0.0001
	‘Xiaguang’	*r*(*y*, *x*)	0.7313	8.0944	0.0001
		*r*(*y*, *x*^2^)	-0.4175	3.4690	0.0010
Firmness without Pericarp	‘Xiahui 8’	*r*(*y*, *x*)	0.7675	9.0390	0.0001
		*r*(*y*, *x*^2^)	-0.6396	6.2818	0.0001
	‘Xiaguang’	*r*(*y*, *x*)	0.6317	6.1522	0.0001
		*r*(*y*, *x*^2^)	-0.3237	2.5828	0.0123

### Model verification

In 2016, after determining the firmness and *I*_AD_ of every fruit for ‘Xiahui 8’ and ‘Xiaguang’, we found that there was still no significant difference between the estimated and observed values. In addition, maturity prediction models for other peach and nectarine varieties were also established using firmness and *I*_AD_ values respectively, and all quadratic polynomial regression equations fitted well.

## Discussion

During the maturation of peach fruit, the internal SSC rises, firmness declines [21,22], the red color appearance increases, and the green color in the pericarp fades [20]. In this study, both ‘Xiahui 8’ and ‘Xiaguang’ had relatively high SSC, a^*^, and a^*^/b^*^ values, and a low fruit firmness at maturity degree II, which indicated that the *I*_AD_ for degree II fruit was lower than that for degree I. The a^*^/b^*^ value can reflect the true color of the fruit [23,24], and was 2.54-fold and 1.94-fold higher in the degree II fruit than degree I ‘Xiahui 8’ and ‘Xiaguang’ fruit, respectively. This is consistent with the opposite change in *I*_AD_, which indicated that the pericarp *I*_AD_ value is closely related to the color of the pericarp. A significant difference in pericarp color, *I*_AD_ value, and most quality indicators was seen in the fruits at the different maturity degree points. This suggested that light absorption and scattering are the main impacting factors on *I*_AD_, which will further affect the pericarp pigment and the change in fruit texture [4,25,26].

The relationship between SSC and fruit maturity is controversial. The SSC prediction for peach fruit by visible/near infrared spectroscopy combined with PLS showed that all the prediction models had a high coefficient of determination, and its prediction accuracy was high for the tested varieties [27,28]. However, Pinto et al. [29] did not observe a significant relationship between SSC and the indicators for maturity. In this study, an extremely significant negative correlation was seen between SSC and *I*_AD_ in ‘Xiahui 8’, but there were no significant positive correlations. Furthermore, there was no correlation for ‘Xiaguang’, which indicated that SSC was a fruit quality indicator but cannot be used to determine harvesting timing [13,30,31]. Previous investigations into the relationship between SSC and fruit maturity showed that it varied depending on the climate zone of the test, the chilling requirements, and the variety tested [8,32].

Soluble sugars and organic acids are important components of soluble solids. In this study, the soluble sugar and organic acid contents were determined when we measured the *I*_AD_, color difference, firmness, and SSC for every fruit of both varieties. There was no positive correlation between SSC and *I*_AD_, but the sorbitol content was closely related to *I*_AD_. The correlation coefficient between sorbitol and *I*_AD_ was 0.35 and 0.71 in ‘Xiahui 8’ and ‘Xiaguang’, respectively, with determination coefficients (*R*^2^) of 0.1225 and 0.5041, respectively. Thus, a prediction model for peach maturity based on the relationship between *I*_AD_ and sorbitol is feasible. Nevertheless, other indicators, such as firmness with or without pericarp, showed a higher correlation coefficient with *I*_AD_ than with sorbitol. Therefore, they may improve the establishment of a stable prediction model.

The establishment of a prediction model for fruit maturity is of great significance when attempting to determine fruit maturity and timely harvesting. It has been demonstrated that a prediction model based on the quality indicators for one variety is more feasible than that based on multiple varieties [1,29,33], but other studies show that the multiple varieties dependent prediction model gives a more accurate determination of fruit maturity [34,35]. The large numbers of different peach varieties mean that the size, color, flesh texture, solids content, mature period, and retention time vary, so it is difficult to create a prediction model that is suitable for all varieties. A prediction model built for a specific variety is more feasible and more accurate. Peach fruit firmness is closely related to harvest maturity, which is the main factor affecting the postharvest storage characteristics of the fruit [36]. Fresh peach market supply and long-distance transport should be combined with fruit hardness when developing an appropriate harvesting system. Gonçalves et al. [13] studied the relationship between fruit firmness and *I*_AD_ using 12 peach varieties and found positive correlations between the firmness without pericarp and *I*_AD_ for all varieties with a minimum *R*^2^ of 0.108 and a maximum *R*^2^ of 0.65. In this study, the *P* value for the linear regression model between fruit firmness with or without pericarp and *I*_AD_ was 0.0001 for both varieties and their Durbin-Watson statistic did not significantly deviate from 2, which indicated that the linear relationship between fruit firmness and *I*_AD_ can be used to predict fruit maturity. However, we also demonstrated that the *R*^2^ of the prediction model had a better quadratic polynomial regression fit between firmness and *I*_AD_. Furthermore, the variance analysis of each quadratic term and linear term showed that their partial regression coefficients reached significant or extremely significant levels, and that the model was more stable than the linear regression model.

Overall, the quadratic polynomial regression method can be used to explore the relationship between fruit quality indicators and maturity degrees. It can also be used to establish a regression equation with good stability and high prediction accuracy, which is of great significance when attempting to determine the maturity of peach fruit and harvest timing. *I*_AD_ values can serve as non-destructive indicators to assay peach maturity, but they have a closer relationship to fruit firmness. The regression relationship between firmness and *I*_AD_ can be used to predict the maturity of other peach and nectarine varieties.

## Supporting information

S1 File Experimental data file. https://figshare.com/s/65769c208399484a1deb.
(DOCX)

## Abbreviations

*I*_AD_: index of absorbance difference
SSC: soluble solids content
PLS: partial least square
L^*^: brightness
C^*^: chroma
H: hue angle
CV: coefficient of variation
