# Supplementary material for: Determination of fruit maturity and its prediction model based on the pericarp index of absorbance difference (IAD) for peaches
Source: PLoS One. 2017 May 15;12(5):e0177511. doi: 10.1371/journal.pone.0177511 (PMC5432102; doi:10.1371/journal.pone.0177511)
Supplement: S1 File — https://figshare.com/s/65769c208399484a1deb. (DOCX) [file pone.0177511.s001.docx]

**Supporting Information File.**

**S1 File. Experimental data file.**

Table A. Raw Data from All Fruit Samples of ‘Xiahui 8’ Peach at Maturity Degree I.

| Fruit number | *I*_AD_ | *L*^*^ | *a*^*^ | *b*^*^ | *C^*^* | *h* | *a*^*^/*b*^*^ | Firmness with Pericarp (N) | Firmness without Pericarp (N) | SSC (°Brix) | Sucrose (g kg^–1^) | Glucose (g kg^–1^) | Fructose (g kg^–1^) | Sorbitol (g kg^–1^) | Malic Acid (g kg^–1^) | Quinic Acid (g kg^–1^) | Citric Acid (g kg^–1^) | Total Sugar (g kg^–1^) | Total Acid (g kg^–1^) | Sugar Acid ratio |
| --- | --- | --- | --- | --- | --- | --- | --- | --- | --- | --- | --- | --- | --- | --- | --- | --- | --- | --- | --- | --- |
| 1 | 0.52 | 68.63 | 18.10 | 21.88 | 28.43 | 50.44 | 0.83 | 193.88 | 109.30 | 12.05 | 46.96 | 12.34 | 10.88 | 6.34 | 4.64 | 2.44 | 0.35 | 76.53 | 7.43 | 10.29 |
| 2 | 0.37 | 77.17 | 4.09 | 25.59 | 25.95 | 80.82 | 0.16 | 162.36 | 92.99 | 13.55 | 55.88 | 13.44 | 11.38 | 9.07 | 2.96 | 2.15 | 0.47 | 89.78 | 5.57 | 16.80 |
| 3 | 0.66 | 68.09 | 16.76 | 23.87 | 30.47 | 56.36 | 0.76 | 179.62 | 83.14 | 10.70 | 44.68 | 13.46 | 12.50 | 2.97 | 2.56 | 1.00 | 0.40 | 73.61 | 3.96 | 18.60 |
| 4 | 0.50 | 70.06 | 13.56 | 23.73 | 27.85 | 61.27 | 0.58 | 166.79 | 98.86 | 11.80 | 49.03 | 13.67 | 12.22 | 7.19 | 2.84 | 1.21 | 0.25 | 82.11 | 4.30 | 19.10 |
| 5 | 0.23 | 65.35 | 23.81 | 20.56 | 31.46 | 40.80 | 1.16 | 140.24 | 72.26 | 11.60 | 54.95 | 6.39 | 9.16 | 4.83 | 2.29 | 0.95 | 0.14 | 75.32 | 3.37 | 22.39 |
| 6 | 0.31 | 65.59 | 21.73 | 20.14 | 29.76 | 43.36 | 1.08 | 183.19 | 92.02 | 12.25 | 53.58 | 15.30 | 13.65 | 7.41 | 2.73 | 1.41 | 0.12 | 89.95 | 4.27 | 21.20 |
| 7 | 0.42 | 73.88 | 10.36 | 24.99 | 28.02 | 69.05 | 0.42 | 145.18 | 85.07 | 11.90 | 45.53 | 14.51 | 13.54 | 5.70 | 2.64 | 1.06 | 0.29 | 79.28 | 3.99 | 19.95 |
| 8 | 0.91 | 66.46 | 16.95 | 23.13 | 29.60 | 55.00 | 0.77 | 154.76 | 101.94 | 9.50 | 33.36 | 13.03 | 12.59 | 2.51 | 2.65 | 0.99 | 0.44 | 61.49 | 4.08 | 15.07 |
| 9 | 0.25 | 76.19 | 5.17 | 25.04 | 25.59 | 78.18 | 0.21 | 178.60 | 95.09 | 12.20 | 49.42 | 13.38 | 12.02 | 6.15 | 2.20 | 0.93 | 0.27 | 80.97 | 3.39 | 23.98 |
| 10 | 0.86 | 66.35 | 18.44 | 25.03 | 31.11 | 53.84 | 0.73 | 154.62 | 76.28 | 10.90 | 35.20 | 14.71 | 15.12 | 2.88 | 2.64 | 1.39 | 0.31 | 67.91 | 4.35 | 15.71 |
| 11 | 0.28 | 74.67 | 10.62 | 24.85 | 27.04 | 67.08 | 0.42 | 195.11 | 106.65 | 13.65 | 60.23 | 13.79 | 11.61 | 9.87 | 2.95 | 0.98 | 0.51 | 95.50 | 4.44 | 21.58 |
| 12 | 0.60 | 61.13 | 24.54 | 23.33 | 34.52 | 43.82 | 1.13 | 185.99 | 98.34 | 11.05 | 46.71 | 16.24 | 15.54 | 5.89 | 2.40 | 1.34 | 0.38 | 84.38 | 4.11 | 20.55 |
| 13 | 0.67 | 62.90 | 21.19 | 21.18 | 30.99 | 47.77 | 1.04 | 160.99 | 100.61 | 11.85 | 57.14 | 14.00 | 12.10 | 6.77 | 2.59 | 1.20 | 0.30 | 90.01 | 4.10 | 22.02 |
| 14 | 0.51 | 71.08 | 17.15 | 22.43 | 28.31 | 52.46 | 0.77 | 164.79 | 90.41 | 11.75 | 51.95 | 13.06 | 11.28 | 7.27 | 2.44 | 0.78 | 0.35 | 83.57 | 3.57 | 23.43 |
| 15 | 0.25 | 74.97 | 10.34 | 24.97 | 27.16 | 68.06 | 0.41 | 185.60 | 104.90 | 13.00 | 58.12 | 15.02 | 13.15 | 7.29 | 2.50 | 0.94 | 0 | 93.59 | 3.44 | 27.27 |
| 16 | 0.82 | 61.16 | 21.72 | 23.07 | 31.70 | 46.68 | 0.94 | 156.05 | 83.06 | 12.55 | 53.98 | 12.69 | 10.75 | 7.94 | 2.16 | 0.92 | 0.26 | 85.36 | 3.34 | 25.72 |
| 17 | 0.42 | 69.00 | 15.14 | 24.72 | 29.00 | 58.62 | 0.61 | 165.73 | 84.40 | 11.85 | 54.39 | 12.69 | 11.04 | 6.64 | 2.79 | 0.84 | 0.28 | 84.76 | 3.90 | 21.76 |
| 18 | 0.44 | 69.88 | 16.17 | 20.49 | 26.19 | 51.75 | 0.80 | 160.09 | 81.24 | 11.65 | 50.15 | 12.68 | 10.99 | 5.18 | 2.33 | 0.75 | 0.39 | 79.00 | 3.48 | 22.73 |
| 19 | 1.17 | 63.40 | 20.08 | 22.70 | 31.16 | 50.08 | 0.92 | 164.88 | 87.12 | 8.75 | 25.89 | 11.59 | 11.48 | 1.18 | 3.50 | 1.04 | 0.55 | 50.14 | 5.09 | 9.86 |
| 20 | 0.59 | 75.06 | 6.35 | 25.09 | 25.89 | 75.72 | 0.25 | 203.85 | 110.95 | 12.50 | 53.05 | 13.81 | 12.08 | 7.35 | 2.44 | 1.33 | 0.40 | 86.30 | 4.17 | 20.72 |
| 21 | 0.44 | 77.68 | 5.27 | 26.92 | 27.50 | 78.91 | 0.20 | 195.36 | 102.45 | 11.85 | 50.44 | 12.84 | 11.12 | 5.63 | 2.61 | 1.08 | 0.47 | 80.02 | 4.16 | 19.24 |
| 22 | 0.19 | 68.32 | 17.56 | 20.21 | 27.69 | 50.88 | 0.91 | 177.73 | 98.78 | 14.15 | 60.56 | 13.73 | 11.29 | 8.54 | 2.64 | 1.24 | 0.54 | 94.11 | 4.43 | 21.26 |
| 23 | 0.48 | 66.55 | 22.16 | 21.11 | 30.70 | 43.82 | 1.06 | 184.57 | 104.32 | 11.85 | 49.34 | 12.46 | 10.89 | 6.09 | 2.56 | 1.15 | 0.41 | 78.78 | 4.13 | 19.08 |
| 24 | 1.00 | 66.30 | 12.64 | 25.82 | 29.35 | 64.08 | 0.51 | 166.28 | 94.45 | 10.65 | 42.33 | 13.57 | 12.67 | 5.79 | 2.64 | 1.08 | 0.42 | 74.35 | 4.14 | 17.93 |
| 25 | 0.56 | 69.54 | 17.98 | 22.15 | 29.00 | 51.63 | 0.84 | 189.79 | 102.78 | 11.30 | 40.33 | 12.71 | 11.87 | 4.47 | 2.72 | 1.33 | 0.38 | 69.37 | 4.43 | 15.67 |
| 26 | 0.40 | 69.33 | 16.46 | 23.44 | 31.61 | 59.07 | 0.80 | 170.47 | 97.82 | 12.10 | 51.94 | 12.93 | 11.51 | 7.42 | 2.67 | 1.40 | 0.41 | 83.81 | 4.48 | 18.73 |
| 27 | 0.99 | 64.24 | 19.12 | 22.59 | 29.86 | 50.47 | 0.85 | 191.24 | 109.50 | 12.15 | 52.08 | 12.53 | 10.66 | 7.21 | 2.81 | 0.80 | 0.29 | 82.48 | 3.90 | 21.24 |
| 28 | 0.48 | 72.23 | 11.25 | 23.04 | 25.64 | 64.00 | 0.49 | 177.83 | 101.84 | 13.10 | 54.70 | 13.11 | 11.30 | 7.42 | 3.03 | 1.23 | 0.47 | 86.53 | 4.74 | 18.26 |
| 29 | 0.34 | 68.82 | 16.99 | 24.26 | 31.50 | 57.21 | 0.77 | 160.09 | 97.10 | 12.65 | 55.03 | 12.95 | 11.29 | 6.15 | 2.67 | 1.32 | 0.47 | 85.41 | 4.46 | 19.17 |
| 30 | 0.20 | 76.74 | 9.22 | 23.00 | 24.97 | 67.87 | 0.41 | 179.13 | 104.11 | 13.20 | 57.74 | 12.82 | 10.53 | 8.28 | 2.62 | 0.99 | 0.26 | 89.38 | 3.87 | 23.28 |

Table B. Raw Data from All Fruit Samples of ‘Xiahui 8’ Peach at Maturity Degree II.

| Fruit number | *I*_AD_ | *L*^*^ | *a*^*^ | *b*^*^ | *C^*^* | *h* | *a*^*^/*b*^*^ | Firmness with Pericarp (N) | Firmness without Pericarp (N) | SSC (°Brix) | Sucrose (g kg^–1^) | Glucose (g kg^–1^) | Fructose (g kg^–1^) | Sorbitol (g kg^–1^) | Malic Acid (g kg^–1^) | Quinic Acid (g kg^–1^) | Citric Acid (g kg^–1^) | Total Sugar (g kg^–1^) | Total Acid (g kg^–1^) | Sugar Acid ratio |
| --- | --- | --- | --- | --- | --- | --- | --- | --- | --- | --- | --- | --- | --- | --- | --- | --- | --- | --- | --- | --- |
| 1 | 0.22 | 48.65 | 32.05 | 16.24 | 35.93 | 26.81 | 1.98 | 131.24 | 59.15 | 12.50 | 58.66 | 14.78 | 12.43 | 3.57 | 2.05 | 1.10 | 0.15 | 89.44 | 3.29 | 27.22 |
| 2 | 0.07 | 50.63 | 35.90 | 19.55 | 40.89 | 28.57 | 1.84 | 52.61 | 9.69 | 10.35 | 43.94 | 13.28 | 12.26 | 0.58 | 1.71 | 0.75 | 0 | 70.06 | 2.46 | 28.49 |
| 3 | 0.23 | 55.23 | 35.70 | 18.90 | 40.39 | 27.90 | 1.89 | 123.51 | 56.97 | 10.75 | 48.05 | 14.33 | 12.84 | 2.28 | 2.02 | 0.56 | 0.11 | 77.50 | 2.70 | 28.86 |
| 4 | 0.12 | 57.15 | 33.73 | 19.01 | 38.74 | 29.45 | 1.78 | 145.14 | 67.32 | 14.40 | 67.36 | 16.40 | 13.70 | 6.80 | 2.18 | 0.93 | 0 | 104.26 | 3.12 | 33.48 |
| 5 | 0.15 | 45.31 | 32.20 | 16.74 | 36.29 | 27.45 | 1.93 | 89.56 | 50.72 | 10.85 | 53.68 | 13.80 | 11.78 | 1.68 | 1.80 | 0.94 | 0 | 80.94 | 2.74 | 29.61 |
| 6 | 0.28 | 53.75 | 34.61 | 19.40 | 39.68 | 29.26 | 1.79 | 136.04 | 66.03 | 13.50 | 54.16 | 14.02 | 11.91 | 1.67 | 1.76 | 0.93 | 0 | 81.76 | 2.68 | 30.46 |
| 7 | 0.12 | 57.69 | 32.28 | 18.28 | 37.11 | 29.55 | 1.77 | 110.28 | 59.61 | 13.50 | 59.88 | 15.91 | 13.71 | 2.61 | 1.99 | 1.13 | 0 | 92.11 | 3.12 | 29.49 |
| 8 | 0.09 | 44.57 | 30.94 | 14.71 | 34.26 | 25.44 | 2.10 | 82.53 | 40.99 | 12.60 | 61.32 | 16.17 | 14.10 | 2.05 | 2.30 | 1.39 | 0 | 93.65 | 3.69 | 25.37 |
| 9 | 0.13 | 59.76 | 31.33 | 21.43 | 38.05 | 34.55 | 1.48 | 124.54 | 64.10 | 10.00 | 57.47 | 14.62 | 12.64 | 2.82 | 2.33 | 0.77 | 0 | 87.55 | 3.10 | 28.30 |
| 10 | 0.05 | 54.15 | 30.53 | 18.78 | 36.02 | 31.47 | 1.70 | 82.79 | 30.89 | 10.50 | 44.92 | 14.74 | 14.12 | 0.72 | 2.03 | 1.01 | 0 | 74.50 | 3.04 | 24.55 |
| 11 | 0.12 | 54.74 | 31.13 | 17.91 | 35.91 | 29.91 | 1.74 | 90.77 | 40.01 | 17.25 | 70.37 | 17.13 | 14.10 | 7.56 | 2.15 | 0.97 | 0 | 109.15 | 3.12 | 34.97 |
| 12 | 0.20 | 49.87 | 32.94 | 19.57 | 38.33 | 30.60 | 1.70 | 67.74 | 23.91 | 11.20 | 44.04 | 15.54 | 15.79 | 0.97 | 2.18 | 0.96 | 0.13 | 76.34 | 3.28 | 23.32 |
| 13 | 0 | 58.66 | 29.61 | 18.37 | 34.85 | 31.89 | 1.61 | 66.54 | 47.25 | 15.05 | 61.30 | 15.28 | 12.94 | 2.83 | 2.13 | 1.13 | 0 | 92.35 | 3.26 | 28.32 |
| 14 | 0.17 | 53.83 | 35.81 | 18.74 | 40.42 | 27.57 | 1.92 | 130.27 | 53.79 | 10.55 | 45.21 | 14.15 | 12.95 | 2.29 | 2.04 | 0.96 | 0 | 74.60 | 3.00 | 24.89 |
| 15 | 0 | 56.07 | 31.72 | 20.20 | 37.60 | 32.48 | 1.57 | 47.35 | 9.82 | 11.90 | 44.27 | 16.56 | 16.39 | 0.91 | 2.11 | 1.29 | 0 | 78.13 | 3.40 | 23.00 |
| 16 | 0.05 | 52.61 | 36.03 | 17.84 | 40.21 | 26.34 | 2.02 | 80.85 | 43.43 | 15.35 | 75.00 | 17.44 | 14.41 | 4.48 | 2.29 | 1.00 | 0 | 111.33 | 3.29 | 33.84 |
| 17 | 0.10 | 53.37 | 26.43 | 19.27 | 33.04 | 36.01 | 1.46 | 45.54 | 9.11 | 13.00 | 48.28 | 17.27 | 18.33 | 1.22 | 2.43 | 1.85 | 0 | 85.11 | 4.28 | 19.94 |
| 18 | 0.33 | 52.10 | 34.99 | 19.46 | 40.07 | 29.03 | 1.81 | 126.23 | 64.36 | 13.05 | 58.32 | 15.42 | 12.93 | 5.61 | 2.42 | 0.87 | 0 | 92.28 | 3.29 | 28.08 |
| 19 | 0.09 | 62.08 | 27.90 | 19.75 | 34.27 | 35.76 | 1.41 | 59.33 | 31.12 | 11.75 | 48.74 | 14.83 | 14.73 | 2.16 | 2.41 | 1.35 | 0.13 | 80.45 | 3.89 | 20.78 |
| 20 | 0.05 | 57.80 | 30.11 | 20.58 | 36.58 | 34.78 | 1.47 | 41.27 | 10.39 | 12.25 | 38.74 | 14.50 | 15.30 | 0.77 | 1.88 | 1.77 | 0 | 69.32 | 3.65 | 18.98 |
| 21 | 0.36 | 42.77 | 30.97 | 16.12 | 34.92 | 27.33 | 1.94 | 131.91 | 44.07 | 10.70 | 45.60 | 15.37 | 14.87 | 2.04 | 2.28 | 1.12 | 0.13 | 77.87 | 3.53 | 22.08 |
| 22 | 0 | 48.05 | 29.65 | 15.42 | 33.41 | 27.43 | 1.93 | 40.01 | 15.00 | 13.55 | 57.42 | 13.09 | 11.86 | 2.01 | 1.78 | 0.85 | 0 | 84.39 | 2.62 | 32.83 |
| 23 | 0.03 | 49.98 | 33.12 | 17.34 | 37.41 | 27.57 | 1.93 | 59.01 | 42.58 | 14.15 | 65.08 | 15.01 | 13.33 | 2.59 | 2.11 | 1.18 | 0 | 96.01 | 3.30 | 29.15 |
| 24 | 0 | 48.55 | 29.88 | 15.70 | 33.76 | 27.54 | 1.92 | 77.97 | 44.59 | 12.45 | 65.62 | 14.88 | 12.07 | 2.95 | 2.21 | 1.09 | 0 | 95.52 | 3.30 | 28.92 |
| 25 | 0 | 58.21 | 26.17 | 17.65 | 31.57 | 34.10 | 1.48 | 54.43 | 20.76 | 11.60 | 47.39 | 12.39 | 10.94 | 1.00 | 1.91 | 1.10 | 0 | 71.72 | 3.01 | 23.86 |
| 26 | 0.03 | 53.28 | 30.14 | 17.34 | 34.82 | 30.03 | 1.75 | 64.48 | 30.47 | 13.50 | 62.24 | 15.30 | 12.85 | 2.12 | 1.96 | 1.16 | 0 | 92.51 | 3.12 | 29.68 |
| 27 | 0.19 | 48.47 | 36.18 | 18.22 | 40.50 | 26.73 | 1.99 | 128.62 | 65.44 | 10.15 | 43.53 | 12.72 | 11.16 | 1.86 | 1.89 | 0.72 | 0 | 69.27 | 2.61 | 26.59 |
| 28 | 0 | 57.11 | 23.11 | 20.12 | 31.27 | 40.80 | 1.28 | 26.60 | 5.97 | 11.95 | 55.41 | 14.90 | 13.94 | 0.65 | 2.10 | 1.49 | 0 | 84.90 | 3.60 | 23.83 |
| 29 | 0.17 | 58.97 | 32.79 | 18.38 | 37.59 | 29.28 | 1.78 | 114.16 | 52.04 | 13.55 | 66.60 | 15.77 | 12.71 | 5.40 | 2.62 | 1.24 | 0 | 100.47 | 3.86 | 26.34 |
| 30 | 0.31 | 54.28 | 30.44 | 18.22 | 35.48 | 30.85 | 1.68 | 138.67 | 61.73 | 11.40 | 52.45 | 13.63 | 11.48 | 3.11 | 2.33 | 0.92 | 0 | 80.67 | 3.25 | 24.85 |

Table C. Raw Data from All Fruit Samples of ‘Xiaguang’ Nectarine at Maturity Degree I.

| Fruit number | *I*_AD_ | *L*^*^ | *a*^*^ | *b*^*^ | *C^*^* | *h* | *a*^*^/*b*^*^ | Firmness with Pericarp (N) | Firmness without Pericarp (N) | SSC (°Brix) | Sucrose (g kg^–1^) | Glucose (g kg^–1^) | Fructose (g kg^–1^) | Sorbitol (g kg^–1^) | Malic Acid (g kg^–1^) | Quinic Acid (g kg^–1^) | Citric Acid (g kg^–1^) | Total Sugar (g kg^–1^) | Total Acid (g kg^–1^) | Sugar Acid ratio |
| --- | --- | --- | --- | --- | --- | --- | --- | --- | --- | --- | --- | --- | --- | --- | --- | --- | --- | --- | --- | --- |
| 1 | 1.18 | 68.76 | 3.00 | 43.47 | 43.62 | 85.95 | 0.07 | 191.88 | 99.52 | 11.80 | 57.79 | 15.14 | 13.25 | 9.39 | 3.66 | 1.63 | 0.49 | 95.57 | 5.77 | 16.55 |
| 2 | 1.19 | 62.30 | 15.80 | 38.21 | 41.35 | 67.55 | 0.41 | 170.87 | 77.80 | 11.30 | 48.67 | 14.26 | 12.50 | 6.77 | 3.29 | 1.34 | 0.53 | 82.19 | 5.17 | 15.92 |
| 3 | 1.01 | 67.84 | 11.32 | 39.96 | 41.80 | 73.83 | 0.29 | 159.30 | 80.43 | 15.55 | 68.29 | 16.67 | 14.02 | 10.53 | 3.63 | 2.43 | 0.64 | 109.51 | 6.69 | 16.37 |
| 4 | 1.52 | 61.60 | 10.26 | 37.91 | 39.28 | 74.90 | 0.27 | 193.85 | 104.43 | 17.10 | 67.05 | 17.30 | 14.02 | 19.24 | 3.39 | 2.02 | 0.51 | 117.61 | 5.92 | 19.88 |
| 5 | 1.20 | 60.07 | 18.91 | 35.29 | 41.86 | 61.48 | 0.61 | 141.39 | 61.56 | 12.35 | 51.89 | 14.77 | 12.30 | 6.96 | 3.08 | 1.71 | 1.34 | 85.92 | 6.13 | 15.1 |
| 6 | 0.60 | 61.27 | 24.08 | 35.56 | 42.95 | 55.93 | 0.68 | 149.47 | 89.74 | 13.10 | 57.85 | 14.71 | 11.36 | 11.29 | 3.39 | 1.30 | 0.27 | 95.21 | 4.96 | 19.26 |
| 7 | 0.75 | 64.27 | 18.62 | 38.36 | 43.63 | 63.24 | 0.53 | 144.19 | 75.59 | 12.65 | 57.03 | 15.73 | 13.10 | 9.63 | 3.08 | 1.40 | 0.42 | 95.48 | 4.90 | 19.50 |
| 8 | 0.52 | 62.03 | 23.19 | 37.42 | 44.60 | 58.43 | 0.64 | 119.94 | 68.06 | 11.90 | 50.69 | 14.53 | 11.90 | 4.71 | 2.78 | 1.15 | 0.40 | 81.82 | 4.34 | 19.11 |
| 9 | 0.98 | 61.78 | 24.31 | 35.31 | 43.09 | 55.54 | 0.70 | 186.88 | 81.65 | 12.40 | 52.15 | 16.34 | 14.36 | 10.04 | 2.97 | 1.75 | 0.53 | 92.89 | 5.24 | 17.75 |
| 10 | 1.04 | 61.86 | 21.99 | 37.83 | 44.07 | 59.76 | 0.59 | 170.84 | 101.77 | 13.05 | 57.87 | 15.59 | 13.14 | 9.60 | 3.03 | 1.89 | 0.53 | 96.20 | 5.46 | 17.63 |
| 11 | 0.82 | 60.26 | 25.81 | 32.58 | 41.63 | 51.49 | 0.80 | 137.06 | 62.41 | 16.70 | 49.67 | 13.90 | 11.64 | 8.15 | 2.56 | 1.48 | 0.47 | 83.36 | 4.50 | 18.57 |
| 12 | 1.40 | 53.75 | 28.59 | 29.64 | 42.29 | 46.56 | 1.05 | 173.54 | 94.25 | 14.45 | 74.80 | 16.72 | 13.48 | 9.89 | 2.81 | 1.98 | 0.25 | 114.89 | 5.04 | 22.95 |
| 13 | 1.21 | 59.00 | 15.84 | 34.61 | 40.89 | 63.78 | 0.59 | 194.54 | 110.06 | 14.15 | 64.42 | 16.65 | 14.00 | 16.61 | 3.74 | 1.75 | 0.48 | 111.68 | 5.97 | 18.76 |
| 14 | 1.02 | 66.13 | 12.84 | 39.16 | 41.22 | 71.86 | 0.33 | 155.11 | 68.87 | 12.75 | 64.31 | 16.66 | 13.56 | 14.72 | 3.26 | 1.85 | 0.46 | 109.26 | 5.57 | 19.67 |
| 15 | 0.82 | 66.68 | 17.11 | 44.08 | 47.29 | 68.73 | 0.39 | 122.38 | 64.65 | 15.15 | 54.74 | 15.42 | 13.30 | 6.09 | 2.54 | 1.69 | 0.42 | 89.55 | 4.64 | 19.30 |
| 16 | 0.92 | 62.90 | 18.93 | 37.00 | 41.78 | 62.93 | 0.52 | 169.65 | 99.78 | 14.80 | 60.40 | 14.61 | 11.93 | 4.47 | 2.84 | 1.46 | 0 | 91.41 | 4.30 | 21.25 |
| 17 | 0.99 | 56.46 | 22.03 | 31.12 | 38.53 | 55.20 | 0.72 | 150.35 | 72.77 | 14.80 | 58.62 | 15.39 | 12.65 | 12.08 | 3.25 | 1.52 | 0.42 | 98.74 | 5.19 | 19.03 |
| 18 | 1.23 | 52.68 | 29.13 | 25.67 | 38.84 | 41.44 | 1.14 | 192.67 | 94.19 | 14.25 | 72.33 | 16.31 | 13.77 | 14.84 | 3.56 | 1.78 | 0.46 | 117.23 | 5.80 | 20.21 |
| 19 | 1.23 | 63.92 | 11.47 | 41.39 | 44.18 | 73.79 | 0.31 | 166.35 | 90.70 | 20.25 | 58.14 | 15.77 | 13.31 | 15.66 | 3.09 | 1.95 | 0.45 | 102.89 | 5.49 | 18.76 |
| 20 | 0.94 | 65.58 | 10.02 | 42.82 | 44.44 | 76.53 | 0.25 | 140.98 | 83.90 | 15.50 | 76.93 | 18.05 | 15.36 | 13.89 | 3.43 | 2.17 | 0.56 | 124.24 | 6.16 | 20.37 |
| 21 | 0.58 | 58.53 | 28.39 | 32.08 | 42.93 | 48.58 | 0.89 | 123.04 | 56.68 | 14.60 | 60.69 | 16.53 | 13.99 | 7.24 | 3.03 | 1.51 | 0.27 | 98.45 | 4.82 | 20.51 |
| 22 | 0.37 | 57.01 | 32.02 | 35.08 | 47.52 | 47.65 | 0.91 | 79.91 | 38.13 | 11.65 | 64.73 | 16.30 | 14.03 | 9.09 | 3.01 | 1.69 | 0.11 | 104.14 | 4.81 | 21.67 |
| 23 | 1.24 | 52.02 | 29.50 | 27.55 | 41.17 | 43.35 | 1.15 | 167.84 | 74.43 | 13.65 | 56.35 | 14.22 | 12.21 | 4.37 | 2.91 | 1.42 | 0.17 | 87.15 | 4.50 | 19.60 |
| 24 | 0.75 | 65.09 | 14.98 | 36.60 | 40.30 | 67.25 | 0.44 | 145.03 | 65.88 | 11.80 | 43.51 | 14.09 | 13.30 | 11.03 | 2.47 | 1.51 | 0.26 | 81.92 | 4.25 | 19.30 |
| 25 | 0.81 | 53.19 | 34.17 | 28.53 | 44.51 | 39.84 | 1.20 | 153.23 | 74.61 | 15.20 | 48.68 | 12.68 | 11.80 | 6.69 | 2.86 | 1.11 | 0.29 | 79.86 | 4.26 | 18.89 |
| 26 | 1.21 | 63.27 | 12.11 | 40.11 | 41.94 | 73.11 | 0.30 | 159.64 | 82.99 | 18.80 | 66.27 | 15.11 | 12.41 | 7.20 | 2.74 | 1.76 | 0 | 100.99 | 4.50 | 22.54 |
| 27 | 0.43 | 56.82 | 28.15 | 31.50 | 42.36 | 48.11 | 0.91 | 110.13 | 52.45 | 12.65 | 58.87 | 14.73 | 12.77 | 6.04 | 2.90 | 1.26 | 0 | 92.41 | 4.16 | 22.31 |
| 28 | 0.30 | 60.97 | 23.51 | 32.19 | 39.89 | 53.78 | 0.73 | 119.84 | 51.62 | 13.00 | 54.94 | 14.18 | 11.78 | 5.04 | 2.84 | 1.06 | 0 | 85.94 | 3.90 | 22.02 |
| 29 | 0.36 | 53.93 | 18.65 | 28.17 | 36.51 | 51.16 | 1.07 | 111.51 | 56.75 | 13.95 | 68.49 | 15.05 | 13.08 | 7.09 | 3.23 | 1.58 | 0 | 103.72 | 4.80 | 21.61 |
| 30 | 0.97 | 65.52 | 13.96 | 38.43 | 41.02 | 69.73 | 0.37 | 166.79 | 88.75 | 16.50 | 72.44 | 16.46 | 13.06 | 16.19 | 3.54 | 2.18 | 0.39 | 118.15 | 6.12 | 19.35 |

Table D. Raw Data from All Fruit Samples of ‘Xiaguang’ Nectarine at Maturity Degree II.

| Fruit number | *I*_AD_ | *L*^*^ | *a*^*^ | *b*^*^ | *C^*^* | *h* | *a*^*^/*b*^*^ | Firmness with Pericarp (N) | Firmness without Pericarp (N) | SSC (°Brix) | Sucrose (g kg^–1^) | Glucose (g kg^–1^) | Fructose (g kg^–1^) | Sorbitol (g kg^–1^) | Malic Acid (g kg^–1^) | Quinic Acid (g kg^–1^) | Citric Acid (g kg^–1^) | Total Sugar (g kg^–1^) | Total Acid (g kg^–1^) | Sugar Acid ratio |
| --- | --- | --- | --- | --- | --- | --- | --- | --- | --- | --- | --- | --- | --- | --- | --- | --- | --- | --- | --- | --- |
| 1 | 0.22 | 51.87 | 37.01 | 28.09 | 46.63 | 37.07 | 1.35 | 83.32 | 33.75 | 15.00 | 71.93 | 17.38 | 14.74 | 8.63 | 2.92 | 2.19 | 0.49 | 112.67 | 5.60 | 20.14 |
| 2 | 0.13 | 59.16 | 25.14 | 33.01 | 41.61 | 52.40 | 0.78 | 89.49 | 40.40 | 14.10 | 65.84 | 13.51 | 10.43 | 3.39 | 2.85 | 1.85 | 0.11 | 93.17 | 4.81 | 19.39 |
| 3 | 0.25 | 55.30 | 24.24 | 37.25 | 44.71 | 57.06 | 0.66 | 57.53 | 11.73 | 18.90 | 86.85 | 16.73 | 13.72 | 4.50 | 3.40 | 2.15 | 0.24 | 121.80 | 5.79 | 21.22 |
| 4 | 0.50 | 52.00 | 33.54 | 27.41 | 43.90 | 39.95 | 1.28 | 97.35 | 31.25 | 15.30 | 58.76 | 13.72 | 11.84 | 3.93 | 2.22 | 1.45 | 0.17 | 88.27 | 3.85 | 23.63 |
| 5 | 0.53 | 51.65 | 33.19 | 27.57 | 43.21 | 39.75 | 1.21 | 99.51 | 41.29 | 14.45 | 57.91 | 15.74 | 13.35 | 10.99 | 2.92 | 1.44 | 0 | 97.98 | 4.36 | 22.56 |
| 6 | 0.77 | 50.69 | 28.43 | 25.17 | 38.04 | 40.98 | 1.16 | 145.21 | 58.91 | 19.55 | 66.72 | 15.75 | 14.05 | 10.33 | 3.05 | 1.96 | 0.39 | 106.85 | 5.40 | 19.79 |
| 7 | 0.79 | 57.59 | 26.90 | 29.97 | 40.47 | 48.37 | 0.91 | 133.58 | 66.35 | 16.05 | 69.87 | 14.64 | 14.18 | 12.08 | 3.22 | 2.07 | 1.04 | 110.77 | 6.33 | 17.60 |
| 8 | 0.24 | 51.47 | 33.06 | 27.21 | 43.22 | 39.19 | 1.29 | 39.39 | 10.71 | 15.05 | 69.52 | 17.06 | 14.48 | 5.90 | 2.64 | 1.76 | 0.70 | 106.96 | 5.09 | 20.99 |
| 9 | 0.05 | 47.14 | 37.62 | 21.61 | 43.42 | 29.73 | 1.76 | 35.45 | 10.76 | 12.50 | 74.01 | 15.01 | 13.59 | 1.06 | 2.46 | 1.65 | 0.60 | 103.67 | 4.71 | 22.06 |
| 10 | 0.18 | 49.95 | 38.43 | 25.54 | 46.21 | 33.70 | 1.52 | 51.62 | 15.10 | 13.10 | 52.36 | 16.62 | 16.67 | 1.87 | 2.53 | 1.91 | 0.91 | 87.52 | 5.35 | 16.36 |
| 11 | 0.68 | 51.28 | 34.89 | 25.86 | 43.73 | 36.54 | 1.40 | 136.09 | 98.48 | 13.90 | 57.29 | 15.84 | 14.74 | 10.22 | 3.24 | 1.74 | 0.90 | 98.09 | 5.87 | 16.77 |
| 12 | 0.36 | 46.65 | 36.50 | 23.80 | 43.74 | 32.81 | 1.59 | 117.49 | 54.89 | 14.25 | 56.73 | 14.50 | 13.52 | 4.09 | 3.54 | 1.29 | 0.93 | 88.85 | 5.76 | 15.41 |
| 13 | 0.05 | 48.61 | 34.04 | 24.84 | 42.35 | 36.27 | 1.40 | 39.40 | 9.57 | 13.95 | 69.71 | 15.94 | 13.72 | 2.97 | 2.80 | 1.78 | 0.63 | 102.33 | 5.22 | 19.84 |
| 14 | 0.59 | 45.48 | 35.13 | 21.21 | 41.26 | 30.74 | 1.76 | 104.52 | 31.44 | 15.65 | 74.04 | 17.49 | 15.57 | 9.77 | 2.97 | 2.10 | 0 | 116.87 | 5.06 | 23.09 |
| 15 | 0.55 | 52.60 | 32.06 | 27.76 | 42.41 | 40.90 | 1.15 | 127.71 | 51.48 | 16.00 | 66.04 | 15.34 | 15.87 | 9.73 | 2.78 | 1.86 | 0.13 | 106.99 | 4.77 | 22.62 |
| 16 | 0.57 | 49.54 | 30.00 | 28.56 | 42.68 | 42.75 | 1.24 | 107.60 | 32.71 | 18.35 | 70.23 | 17.12 | 15.80 | 8.95 | 3.22 | 1.69 | 0.29 | 112.10 | 5.21 | 21.55 |
| 17 | 0.06 | 47.52 | 39.33 | 24.57 | 46.46 | 31.90 | 1.63 | 32.80 | 9.46 | 14.05 | 64.16 | 10.55 | 13.08 | 1.43 | 2.46 | 1.44 | 0.29 | 89.23 | 4.19 | 21.48 |
| 18 | 0.29 | 52.34 | 33.21 | 25.12 | 41.81 | 37.04 | 1.36 | 48.14 | 14.22 | 11.25 | 44.47 | 12.41 | 11.89 | 2.09 | 3.68 | 1.24 | 0.50 | 70.86 | 5.41 | 13.09 |
| 19 | 0 | 52.19 | 33.76 | 26.50 | 43.42 | 37.64 | 1.38 | 57.54 | 5.43 | 15.20 | 52.63 | 13.25 | 13.44 | 2.05 | 2.27 | 1.37 | 0.16 | 81.37 | 3.80 | 21.67 |
| 20 | 1.03 | 52.59 | 27.30 | 26.00 | 37.87 | 43.56 | 1.07 | 164.53 | 77.37 | 16.60 | 63.59 | 15.43 | 14.98 | 10.91 | 3.08 | 2.05 | 0.53 | 104.92 | 5.66 | 18.57 |
| 21 | 0.64 | 54.99 | 31.06 | 29.12 | 42.66 | 43.03 | 1.08 | 141.59 | 66.48 | 17.45 | 76.14 | 16.93 | 14.75 | 13.13 | 4.23 | 1.98 | 0.64 | 120.95 | 6.85 | 17.73 |
| 22 | 0.42 | 58.36 | 29.78 | 32.62 | 44.87 | 47.77 | 0.96 | 86.75 | 27.32 | 16.30 | 74.04 | 15.78 | 14.05 | 6.31 | 3.70 | 1.68 | 0.57 | 110.18 | 5.96 | 18.55 |
| 23 | 0.23 | 56.18 | 31.84 | 30.93 | 44.45 | 44.21 | 1.03 | 90.20 | 25.79 | 13.25 | 64.61 | 14.87 | 13.06 | 1.98 | 3.34 | 1.70 | 0.55 | 94.52 | 5.58 | 16.93 |
| 24 | 0.25 | 54.83 | 30.96 | 28.16 | 41.91 | 42.47 | 1.10 | 54.09 | 15.53 | 10.10 | 29.46 | 13.87 | 14.11 | 0.87 | 3.60 | 1.41 | 0.61 | 58.31 | 5.62 | 10.39 |
| 25 | 0.21 | 50.31 | 33.15 | 27.14 | 43.02 | 39.14 | 1.25 | 61.58 | 6.16 | 16.15 | 67.08 | 15.35 | 15.51 | 4.57 | 3.30 | 2.03 | 0.60 | 102.51 | 5.92 | 17.32 |
| 26 | 0.31 | 58.76 | 22.70 | 32.93 | 41.17 | 54.20 | 0.79 | 89.96 | 36.74 | 18.85 | 81.29 | 16.55 | 16.27 | 6.30 | 3.78 | 1.80 | 0.57 | 120.40 | 6.15 | 19.59 |
| 27 | 0.36 | 62.00 | 22.58 | 34.22 | 41.40 | 56.70 | 0.68 | 100.69 | 35.59 | 17.05 | 81.01 | 15.77 | 15.49 | 4.21 | 3.89 | 2.10 | 0.58 | 116.48 | 6.57 | 17.76 |
| 28 | 0.86 | 51.16 | 31.45 | 24.99 | 40.17 | 38.41 | 1.26 | 183.90 | 85.73 | 15.00 | 67.57 | 16.06 | 16.12 | 13.40 | 3.41 | 2.06 | 0.54 | 113.16 | 6.01 | 18.92 |
| 29 | 0.34 | 55.92 | 30.08 | 29.94 | 42.57 | 44.70 | 1.02 | 62.86 | 10.86 | 15.10 | 74.15 | 12.52 | 16.34 | 4.78 | 2.93 | 2.16 | 0.70 | 107.78 | 5.78 | 18.71 |
| 30 | 0.29 | 47.76 | 36.92 | 22.55 | 43.27 | 31.36 | 1.64 | 53.28 | 17.21 | 13.15 | 57.64 | 14.40 | 13.92 | 4.58 | 2.69 | 2.20 | 0.68 | 90.54 | 5.56 | 16.51 |
